# Untargeted plasma metabolome identifies biomarkers in patients with extracranial arteriovenous malformations

**DOI:** 10.3389/fphys.2023.1207390

**Published:** 2023-09-01

**Authors:** Xueqiang Fan, Xixi Gao, Yisen Deng, Bo Ma, Jingwen Liu, Zhaohua Zhang, Dingkai Zhang, Yuguang Yang, Cheng Wang, Bin He, Qiangqiang Nie, Zhidong Ye, Peng Liu, Jianyan Wen

**Affiliations:** ^1^ Department of Cardiovascular Surgery, China-Japan Friendship Hospital, Beijing, China; ^2^ Graduate School of Peking Union Medical College, Beijing, China; ^3^ Department of Pediatrics, Herman B Wells Center for Pediatric Research, Indiana University School of Medicine, Indianapolis, IN, United States

**Keywords:** arteriovenous malformations, metabolomic, untargeted, plasma, biomarkers

## Abstract

**Objective:** This study aimed to investigate the plasma metabolic profile of patients with extracranial arteriovenous malformations (AVM).

**Method:** Plasma samples were collected from 32 AVM patients and 30 healthy controls (HC). Ultra-high performance liquid chromatography-mass spectrometry (UHPLC-MS) was employed to analyze the metabolic profiles of both groups. Metabolic pathway enrichment analysis was performed through Kyoto Encyclopedia of Genes and Genomes (KEGG) database and MetaboAnalyst. Additionally, machine learning algorithms such as Least Absolute Shrinkage and Selection Operator (LASSO) and random forest (RF) were conducted to screen characteristic metabolites. The effectiveness of the serum biomarkers for AVM was evaluated using a receiver-operating characteristics (ROC) curve.

**Result:** In total, 184 differential metabolites were screened in this study, with 110 metabolites in positive ion mode and 74 metabolites in negative mode. Lipids and lipid-like molecules were the predominant metabolites detected in both positive and negative ion modes. Several significant metabolic pathways were enriched in AVMs, including lipid metabolism, amino acid metabolism, carbohydrate metabolism, and protein translation. Through machine learning algorithms, nine metabolites were identify as characteristic metabolites, including hydroxy-proline, L-2-Amino-4-methylenepentanedioic acid, piperettine, 20-hydroxy-PGF2a, 2,2,4,4-tetramethyl-6-(1-oxobutyl)-1,3,5-cyclohexanetrione, DL-tryptophan, 9-oxoODE, alpha-Linolenic acid, and dihydrojasmonic acid.

**Conclusion:** Patients with extracranial AVMs exhibited significantly altered metabolic patterns compared to healthy controls, which could be identified using plasma metabolomics. These findings suggest that metabolomic profiling can aid in the understanding of AVM pathophysiology and potentially inform clinical diagnosis and treatment.

## Introduction

Arteriovenous malformation (AVM) is a rare congenital vascular malformation characterized by abnormal development of the vascular system at the embryonic stage, resulting in direct anastomosis of arteries and veins to form a tortuous and dilated vascular mass instead of a normal capillary bed (Wassef et al., 2015). The incidence of extracranial AVMs is low and typically diagnosed at birth with no gender difference. The exact etiology and underlying pathogenesis of AVMs remain unclear. Lesions are commonly found in the head and neck (Kohout et al., 1998), followed by limbs, trunk, and viscera, presenting as skin erythema, high skin temperature, palpable pulsation, or tremor. Additional symptoms may include local pain, ulcers, or repeated bleeding, which can lead to heart failure due to hemodynamic abnormalities in severe cases. AVMs can also cause appearance malformations, compression of important tissues and organs, and organ dysfunction. Most AVMs can be diagnosed by clinical manifestations. However, in some cases, digital subtraction angiography (DSA) is required as the gold standard for diagnosis and to guide interventional therapy. Unfortunately, there are no specific laboratory diagnostic indices available for AVMs. Treatment is often challenging and associated with a high recurrence rate (Liu et al., 2010). Available treatment options include interventional embolization therapy, anhydrous ethanol interventional therapy, surgery, and combined therapy. However, multiple and repeated measures are often required (Greene and Orbach, 2011), and there is no mature medical drug treatment available.

Metabolomics is an emerging field of study in the omics discipline focusing on systematically analyzing small molecules in human or animal organisms (Nicholson et al., 1999). This approach aims to identify and quantify a variety of metabolites such as amino acids, fatty acids, carbohydrates, and biochemical intermediates. The ultimate goal of metabolomics is to comprehensively investigate low molecular metabolites in organisms. Metabolites are downstream products of the genome, transcriptome, and proteome, and therefore reflect the end products of gene expression. There are two strategies in metabolomic analysis: targeted and untargeted. Targeted studies focus on predefined metabolites, whereas untargeted research aims to analyze all metabolites as a whole. This latter approach is particularly useful when comparing two groups of subjects, such as healthy individuals and patients with diseases, or between two patients with different diseases. By measuring all metabolites in body fluids, researchers can identify differences between the two groups and search for disease biomarkers. This may lead to better diagnostic methods for clinical uses.

Recent studies have used metabolomic analysis to assess the metabolic profile of vascular diseases and identify relevant biomarkers and major metabolic pathways. This approach may improve our understanding of these diseases and assist in development of new therapeutic targets. Previous metabolomics research in the vascular field has largely focused on cerebrovascular diseases, atherosclerosis, myocardial infarction, heart failure, aortic aneurysms, and aortic dissection. One untargeted metabolomics study discovered phenylacetylglutamine (PAGln) and demonstrated its association with cardiovascular disease and major adverse cardiovascular events (myocardial infarction, stroke, or death) in an independent cohort (*n* = 4,000 subjects) (Nemet et al., 2020). Zhang et al. (2018) identified 36 differential metabolites related to coronary artery disease progression including 15 amino acids, 12 free fatty acids, 8 organic acids, and 1 sialic acid in serum using untargeted metabolomics in 2,324 patients who underwent coronary arteriography from 4 independent centers. A targeted metabolomic analysis and functional metabolomics strategy then revealed that N-Acetylneuraminic acid acts as a potential metabolic marker for coronary artery disease progression. Targeting N-acetylneuraminic acid and its regulatory enzyme neuraminidase-1 may potentially serve as new avenues for therapeutic intervention into myocardial ischemia injury. Razavi et al. (2020) conducted untargeted metabolomic analysis of fasting serum samples in 1,050 white and black participants and identified eight metabolites robustly associated with left ventricular diastolic functions. Among them were formiminoglutamate, 1-methyl-histidine, N2, N5-diacetylornithine, N-trimethyl 5-aminovalerate, N-formylmethionine, 5-methylthioadenosine, and methionine sulfoxide, which were positively associated with left ventricular filling pressure, while butyryl carnitine had a significant positive association with isovolumic relaxation time. It has been reported that plasma succinate concentrations were increased in patients with aortic aneurysm and dissection using untargeted metabolomics and targeted mass spectrometry (Cui et al., 2021). This suggests that plasma succinate concentrations can be used as a biomarker for pre-hospital aortic disease diagnosis and reliable differentiation from acute myocardial infarction and pulmonary embolism when patients present with chest pain. Glycocholic acid has been identified as a potential plasma metabolite marker for the functional outcome after acute ischemic stroke (AIS) in an untargeted LC/MS metabolomics study (Wu et al., 2023). Elevated levels of 4-hydroxyphenylacetic acid and decreased levels of threonine were found to be associated with an increased risk of cardiovascular events (Ferreira-Divino et al., 2022). The levels of circulating acylcarnitines’ level indicate the severity of coronary artery disease (CAD) and may have implications for stratifying patients in future strategies (Gander et al., 2021). These findings highlight significant differences in metabolites between patients with vascular diseases and normal groups, indicating the potential of metabolomics research methods to identify metabolites and enable early diagnosis, targeted therapy, and improved prognosis of vascular diseases.

However, there has been no previous research using metabolomics to analyze changes in metabolites and metabolic pathways in the plasma of patients with AVMs. Therefore, our study aimed to analyze the plasma metabolic profile of patients with extracranial AVMs. We used ultra-high performance liquid chromatography-mass spectrometry (UHPLC-MS) to analyze the plasma metabolic profile between patients with extracranial AVMs and healthy controls (HC) (Mullish et al., 2022). In addition, we employed machine learning algorithms to detect potential biomarkers. Our findings may help identify new targets for therapeutic intervention and improve patient outcomes.

## Materials and methods

### Participants

**FIGURE 1:**
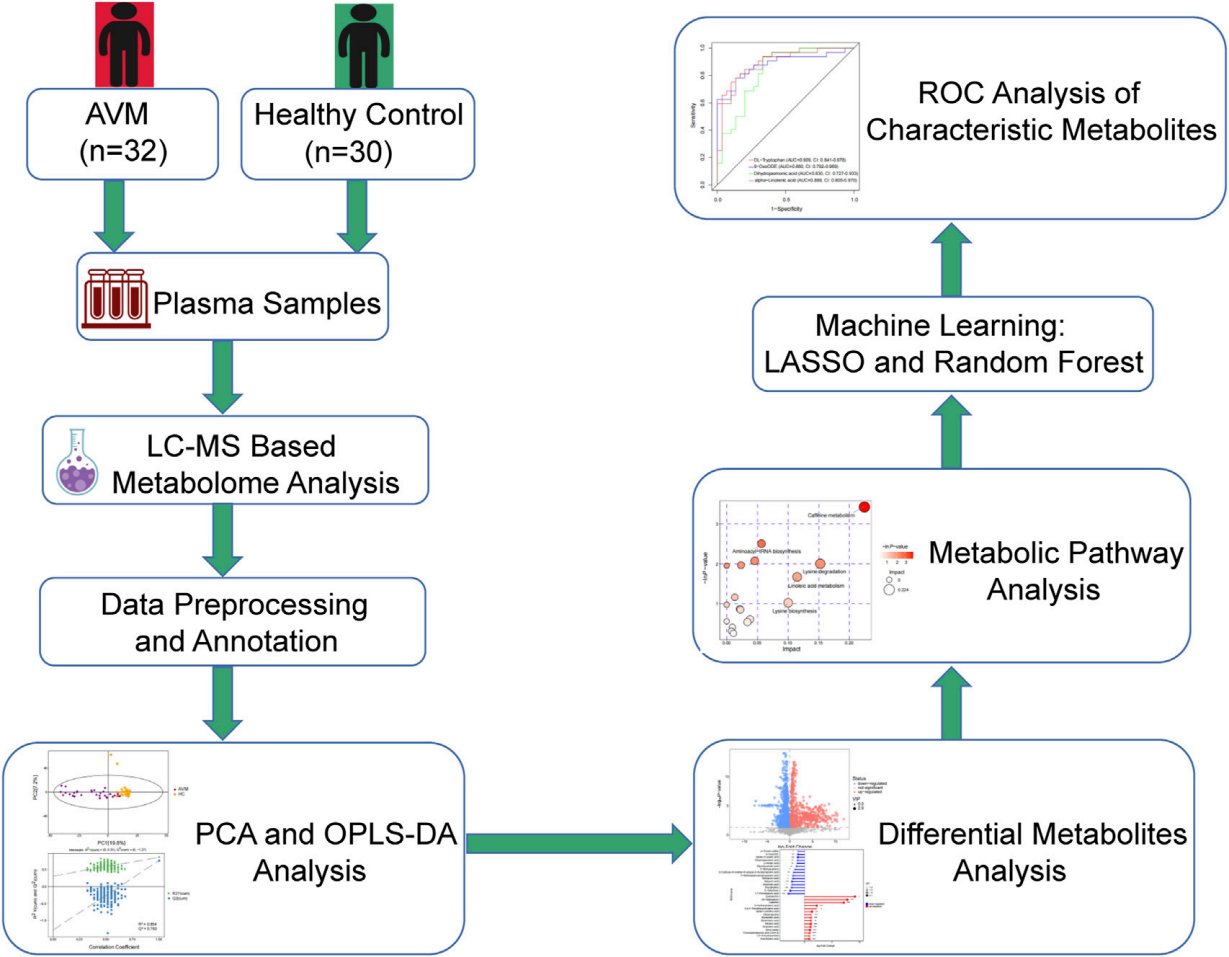
Flow chart of the study as a whole.

This study included 32 patients with extracranial AVM who were recruited from China-Japan Friendship Hospital (Beijing, China) between July 2021 and November 2022. The diagnosis of AVM was based on the protocol formulated by the International Society for the Study of Vascular Anomalies (ISSVA) (Wassef et al., 2015). Exclusion criteria included individuals with: 1) other types of vascular malformations, such as venous malformations or lymphatic malformations; 2) hemangioma; 3) relevant treatment 4 weeks before operation; 4) inflammatory, septic, or autoimmune diseases; 5) age over 75 years; 6) severe dysfunction of heart, liver, kidney, or other organs; 7) a history of malignancy; 8) pregnancy or lactation. Thirty healthy volunteers with no evidence of vascular malformations or metabolic syndrome were enrolled as healthy controls. This study was approved by the Ethics Committee of China-Japan Friendship Hospital, and informed consent was signed by all study participants or their guardians before collecting blood samples. The research adhered to the principles of the Declaration of Helsinki. The flow chart of this study is shown in Figure 1.

### Plasma sample collection and preparation

Peripheral venous blood samples were collected from patients prior to their operation, while blood samples from healthy controls were collected in the morning. Fresh EDTA anti-coagulated blood was centrifuged at 3,000 rpm for 10 min at room temperature. The resulting plasma was immediately stored at −80°C until analysis. Prior to analysis, the blood samples were thawed at 4°C, and 100 μL of the sample was transferred to an EP tube. After the addition of 300 μL of extract solution (methanol, containing isotopically labelled internal standard mixture), the samples were vortexed for 30 s and sonicated for 10 min in an ice-water bath. The internal standard (IS) mixture of the negative ion mode (NEG) comprised L-phenylalanine-D5, decanoic acid-D19, and L-2-chlorophenylalanine. The positive ion mode (POS) internal standard mixture comprised L-phenylalanine-D5, L-2-chlorophenylalanine, and diisobutyl phthalate-D4. Finally, the samples were incubated for 1 h at −40°C to precipitate proteins. Samples were then centrifuged at 12,000 rpm (RCF = 13,800(×g), R = 8.6 cm) for 15 min at 4°C. The resulting supernatant was transferred to a new glass vial for analysis. To prepare the quality control (QC) sample, an equal aliquot of the supernatants from all of the samples was mixed (Alseekh et al., 2021).

### LC-MS/MS analysis

Liquid chromatography-mass spectrometry (LC-MS/MS) analysis were performed using a UHPLC system (Vanquish, Thermo Fisher Scientific) with a UPLC HSS T3 column (2.1 mm × 100 mm, 1.8 μm) coupled to Orbitrap Exploris 120 mass spectrometer (Orbitrap MS, Thermo). The mobile phase consisted of 5 mmol/L ammonium acetate and 5 mmol/L acetic acid in water (A) and acetonitrile (B). The autosampler temperature was set to 4°C, and the injection volume was 2 μL. The Orbitrap Exploris 120 mass spectrometer was chosen for its ability to acquire MS/MS spectra on information-dependent acquisition (IDA) mode in the control of the acquisition software (Xcalibur, Thermo). In this mode, the acquisition software continuously evaluates the full scan MS spectrum. The ESI source conditions were set as follows: sheath gas flow rate as 50 Arb, Aux gas flow rate as 15 Arb, capillary temperature 320°C, full MS resolution as 60,000, MS/MS resolution as 15,000 collision energy as 10/30/60 in NCE mode, spray Voltage as 3.8 kV (positive) or −3.4 kV (negative), respectively (Dunn et al., 2011).

### Data preprocessing and annotation

The raw data underwent conversion to a mzXML format using ProteoWizard and were subsequently processed with an in-house program, which was designed using R and based on XCMS (Smith et al., 2006). This program facilitated peak detection, extraction, alignment, and integration. The extracted data were annotated using an in-house MS2 database (BiotreeDB) with a 0.3 cutoff for annotation, which means that only annotations with a similarity score equal to or higher than 0.3 are considered for further analysis. (Cai et al., 2015). XCMS was used with the centWave method, employing a ppm of 10 and peak widths ranging from 5 to 20. The signal-to-noise ratio (SN) threshold was set at 3. As a pre-filtering step, only peaks with at least 3 intensity values greater than or equal to 1,000 were retained. The function used to calculate the chromatographic peak mz center was wMean, which represents the intensity-weighted average of the peak’s mz values. A minimum m/z dimension difference of −0.001 was required for peaks with overlapping retention times (Supplementary Table S1). The marking value of metabolites is calculated based on Euclidean distance and dot product algorithm, which improves the accuracy of mass spectrum annotation.

### PCA and OPLS-DA analysis

The normalization was performed using an internal standard. The derived data was then log-converted and CTR formatted using SIMCA software (V16.0.2, Sartorius Stedim Data Analytics AB, Umea, Sweden), followed by automatic modeling analysis (Saccenti et al., 2014). This software analysis included both unsupervised principal component analysis (PCA) and supervised orthogonal projections to latent structures-discriminant analysis (OPLS-DA). Following this, the validity of the model was assessed via cross-validation of R^2^Y (the interpretability of the model to the categorical variable Y) and Q^2^ (the predictability of the model). Additionally, a permutation test was conducted, involving the random reordering of the categorical variable Y multiple times (*n* = 200) to generate various random Q^2^ values. This assisted in further testing the validity of the model. *R* ^2^ and Q^2^ represent the model’s interpretability and predictability, respectively, and can be used to assess the model’s performance. In theory, higher values of *R* ^2^ and Q^2^, closer to 1, indicate better model performance, while lower values suggest poorer fitting accuracy. Generally, *R* ^2^ and Q^2^ values above 0.5 are considered good, and values above 0.4 are acceptable. Outside of multivariate statistical methods, the significance of metabolite changes at the univariate level was evaluated using a Student’s t-test. The contribution of variables in the OPLS-DA model was summarized via calculation of the variable importance in the projection (VIP) value.

### Bioinformatics analysis

Metabolites exhibiting VIP values > 1 and *p*-values<0.05 were identified as significantly altered metabolites. The metabolic regulations of these metabolites were visualized using a volcano plot. To perform pathway enrichment analysis, both enrichment analysis and topological analysis were conducted using the Kyoto Encyclopedia of Genes and Genomes (KEGG) database (http://www.kegg.jp/kegg/pathway.html) and MetaboAnalyst (Kanehisa and Goto, 2000; Xia et al., 2015; Kanehisa et al., 2016). Characteristic metabolites were screened using two machine learning algorithms: Least Absolute Shrinkage and Selection Operator (LASSO) analysis and random forest (RF). Potential biomarkers were assessed using receiver operating characteristic (ROC) curve analysis, and the sensitivity and specificity were evaluated by calculating the area under the curve (AUC).

### Statistical analysis

Statistical analysis in this study was performed using SPSS (Version 26.0) and R software (Version 4.2.0). Continuous variables were presented as mean ± standard deviation (SD). For normally distributed data, the Student’s t-test was used for analysis. Categorical variables were described as number (percentage) and compared using the Chi-square test or Fisher’s exact test. A two-tailed *p*-value <0.05 was considered statistically significant.

## Results

### Clinical characteristics of AVM patients and healthy controls

To compare the differences in venous plasma metabolites between extracranial AVM patients and the healthy population, 32 AVM patients and 30 healthy controls were included in this study. The basic characteristics of the sampled population are shown in Supplementary Table S2. There were no differences present between groups with respect to age, gender, body mass index (BMI), and basic diseases such as metabolic syndrome, autoimmune diseases, severe heart, liver or kidney diseases, or malignant tumors.

### Process quality control and data quality control

The stability of the retention time and response strength of the internal standard in quality control (QC) samples was confirmed, indicating effective control of substance residue and cross-contamination between samples within controllable limits. Furthermore, no detectable peaks originating from internal standards were detected in the blank samples. The aggregation of QC samples in the two-dimensional PCA score map was satisfactory, demonstrating high reproducibility (Supplementary Figures S1A, B). Additionally, all QC samples were within ±2 standard deviations in the PCA one-dimensional distribution plot, confirming the high quality of the experimental data (Supplementary Figures S2A, B). In summary, the study exhibited excellent sample quality, experimental methods, and system stability. Consequently, the experimental data were stable and reliable, capable of revealing significant differences in metabolomics between different groups.

### PCA and OPLS-DA analysis

**FIGURE 2:**
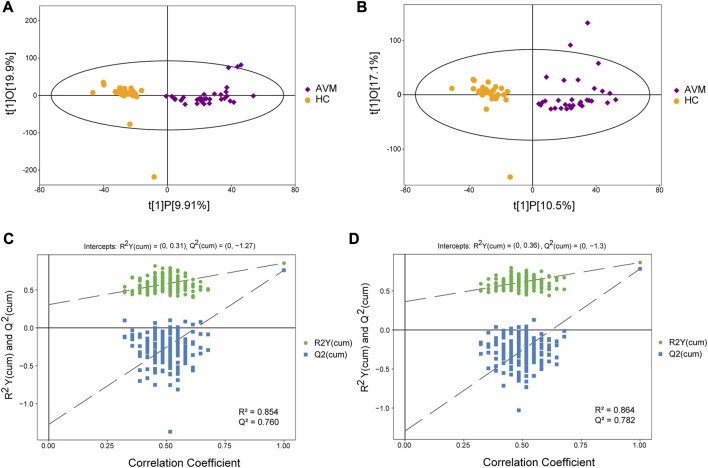
Score scatter plot of the OPLS-DA model for AVM vs. HC group in positive **(A)** and negative **(B)** ion mode. Permutation plot test of OPLS-DA model for group AVM vs. HC in positive **(C)** and negative **(D)** ion mode.

After performing various data preprocessing techniques such as filtering for deviation values, missing values, imputing missing values, and standardizing the data, the raw data were normalized. LC-MS was used to identify a total of 8,741 metabolites in the positive ion mode (POS) and 8,385 metabolites in the negative ion mode (NEG). These metabolites were classified and counted based on their chemical classification information (Supplementary Figures S3A, B). The resulting PCA score map indicated clear clustering between the AVM group and control group (Supplementary Figures S4A, B). It was observed that samples from both groups were mostly within the 95% confidence interval, however, there were significant differences in metabolites between the groups. An OPLS-DA model analysis was conducted on the first principal component, which showed that AVM patients had statistically distinct plasma metabolic profiles compared to the healthy controls (Figures 2A–D). To evaluate the model, permutation analysis was performed, and the results confirmed that the model was both valid and predictive.

### Differential metabolites analysis

**FIGURE 3:**
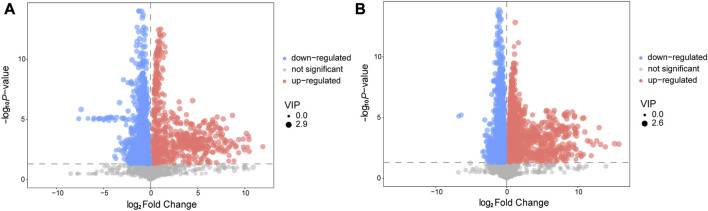
Volcano plot for AVM vs. HC group in positive **(A)** and negative **(B)** ion mode. The abscissa represents the log2(Fold Change) of the substance, the ordinate represents the *p*-value of the Student‘s *t*-test. The scatter represents the VIP value. Significantly upregulated metabolites are indicated in red, significantly downregulated metabolites are indicated in blue, and non-significantly different metabolites are indicated in grey.

We utilized a combination of multivariate and univariate statistical analysis to identify differential metabolite expression between groups. To determine the significance of the results, we considered metabolites with VIP scores >1 and *p*-values< 0.05 as differential metabolites. The differential metabolites were then visualized using a volcano plot (Figures 3A, B). In the positive ion mode, a total of 110 differential metabolites were identified, of which 17 were upregulated and 93 were downregulated. The names of the substances were obtained through a qualitative matching analysis of the secondary mass spectrum (e.g., MS2 name). In the negative ion mode, 74 differential metabolites were identified, with 19 upregulated and 55 downregulated (Supplementary Figure S5B).

### Pathway analysis of differential metabolites

**FIGURE 4:**
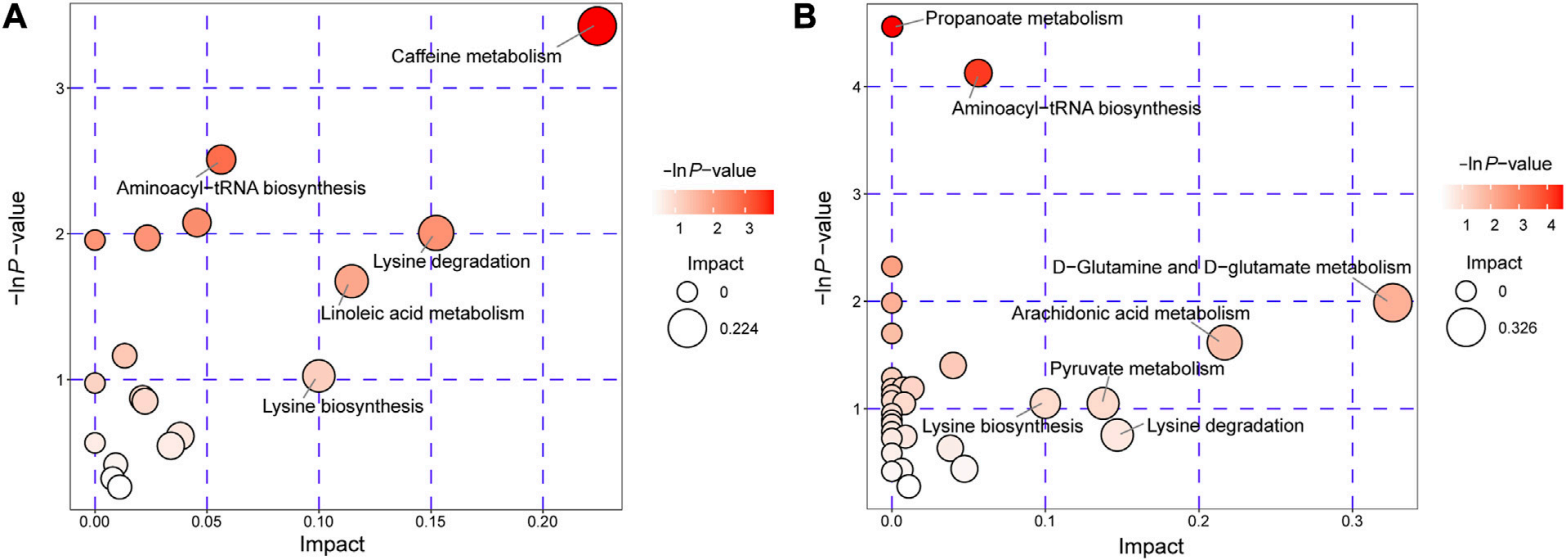
Pathway analysis for AVM vs. HC group in positive ion mode **(A)** and negative ion mode **(B)**. The abscissa and the size of the bubble represent the size of the influence factor. The ordinate and the color of the bubble represent the *p*-value of the enrichment analysis.

We conducted an extensive analysis of differential metabolites authoritative metabolite databases such as KEGG and PubChem. We then searched and analyzed the metabolic pathway database of corresponding species *Homo sapiens* (human) to map these differential metabolites. Our results of the metabolic pathway analysis are displayed in a bubble plot (Figure 4), where we labeled important pathways after performing enrichment and topological analyses. These findings suggest significant alterations in several metabolic pathways, including caffeine metabolism, aminoacyl-tRNA biosynthesis, linoleic acid metabolism, propanoate metabolism, D-glutamine and D-glutamate metabolism, arachidonic acid metabolism, pyruvate metabolism, lysine degradation, and lysine biosynthesis. Protein translation, lipid metabolism, and amino acid metabolism were identified as the main metabolic features in AVM. After obtaining the matching information of differential metabolites, we conducted pathway search and regulatory interaction network analysis to capture the intersection between metabolic pathways and identify potential enzymes and metabolites. This analysis allowed us to investigate how perturbations propagate at the pathway level and how pathways influence each other. The results of the regulatory analysis are visually presented in a network plot, highlighting the interconnectedness and regulatory relationships among the pathways (Supplementary Figures S6A, B).

### Biomarkers from serum for AVM disease

**FIGURE 5:**
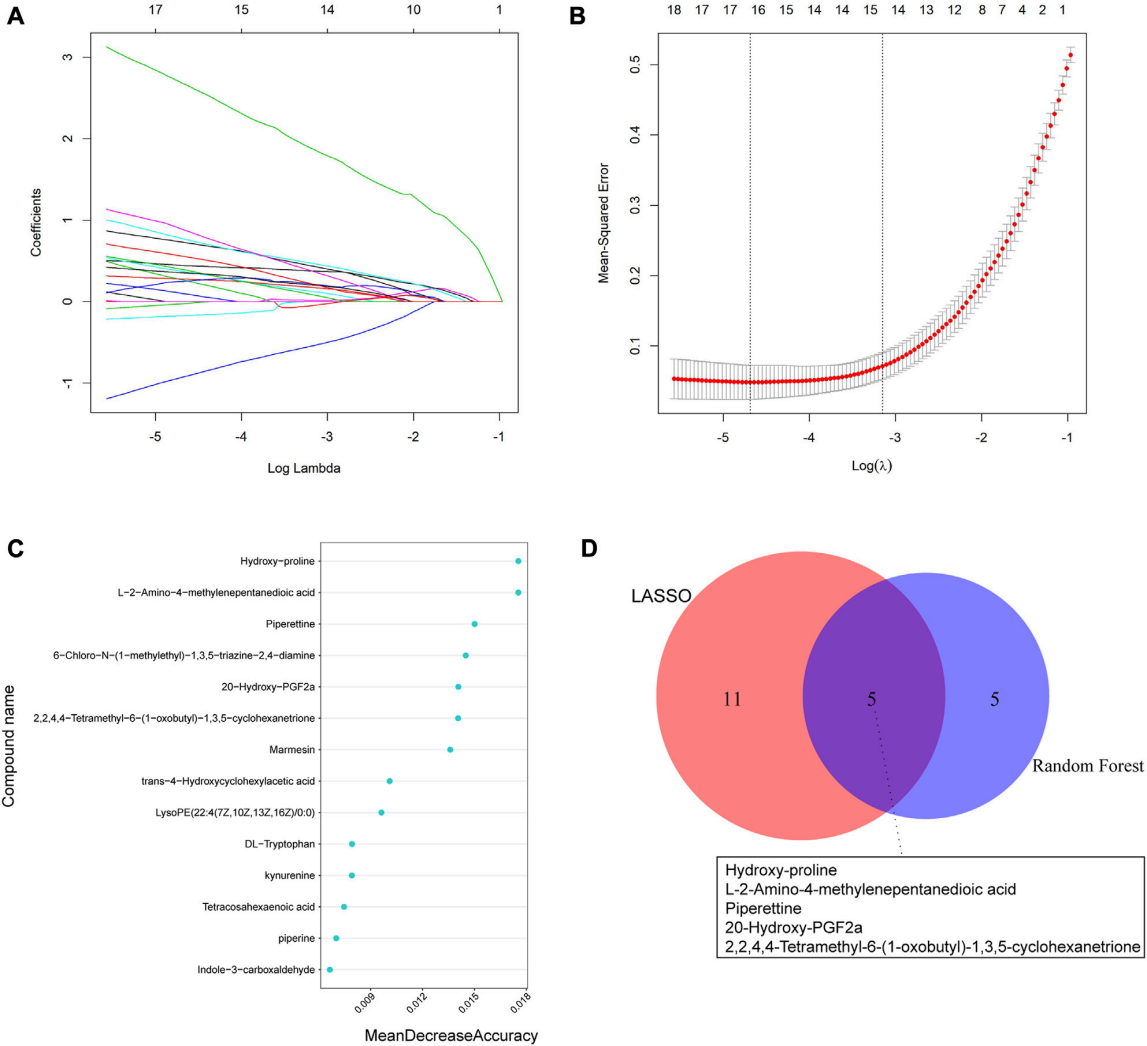
Machine learning algorithms were used to screen characteristic metabolites in the positive ion mode. **(A)** Coefficient profiles of the metabolites in the LASSO model. **(B)** A total of 16 non-zero coefficients were obtained using optimal lambda (λ). **(C)** The top 10 characteristic differential metabolites of the random forest model in the positive ion mode. **(D)** Venn diagram of characteristic metabolites obtained by the two models.

**FIGURE 6:**
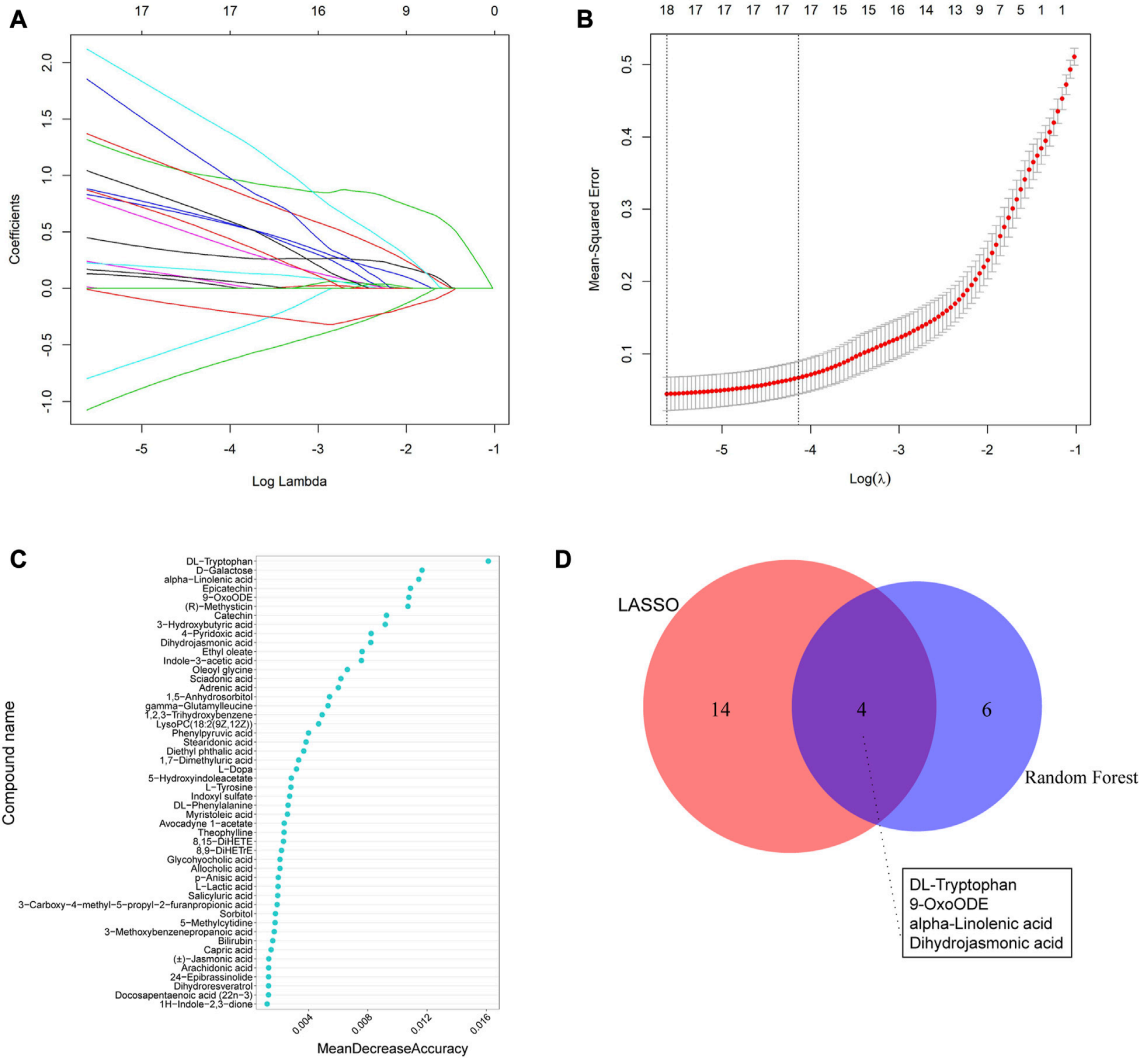
Machine learning methods to screen characteristic metabolites in the negative ion mode. **(A)** Coefficient profiles of the metabolites in the LASSO model. **(B)** A total of 18 non-zero coefficients were obtained using optimal lambda (λ). **(C)** The top 10 characteristic differential metabolites of the random forest model in the positive ion mode. **(D)** Venn diagram of characteristic metabolites obtained from the two models.

We utilized two machine learning techniques, LASSO and random forest, to identify specific differential metabolites. In the positive ion mode, LASSO identified a total of 16 differential metabolites (Figures 5A, B), while the RF model ranked the variables by their mean decrease accuracy (Figure 5C) and the top 10 metabolites were selected as characteristic differential metabolites. Through intersection of the two models, we constructed a Venn diagram (Figure 5D), representing a total of 5 common differential metabolites: hydroxy-proline, L-2-Amino-4-methylenepentanedioic acid, piperettine, 20-hydroxy-PGF2a, and 2,2,4,4-tetramethyl-6-(1-oxobutyl)-1,3,5-cyclohexanetrione. Similarly, we applied the same algorithms in the negative ion mode (Figure 6) and identified 18 differential metabolites using LASSO. After intersecting with the RF model, 4 common differential metabolites were identified: DL-tryptophan, 9-oxoODE, alpha-Linolenic acid, and dihydrojasmonic acid.

### Discriminative ability of serum biomarkers for AVM disease

**FIGURE 7:**
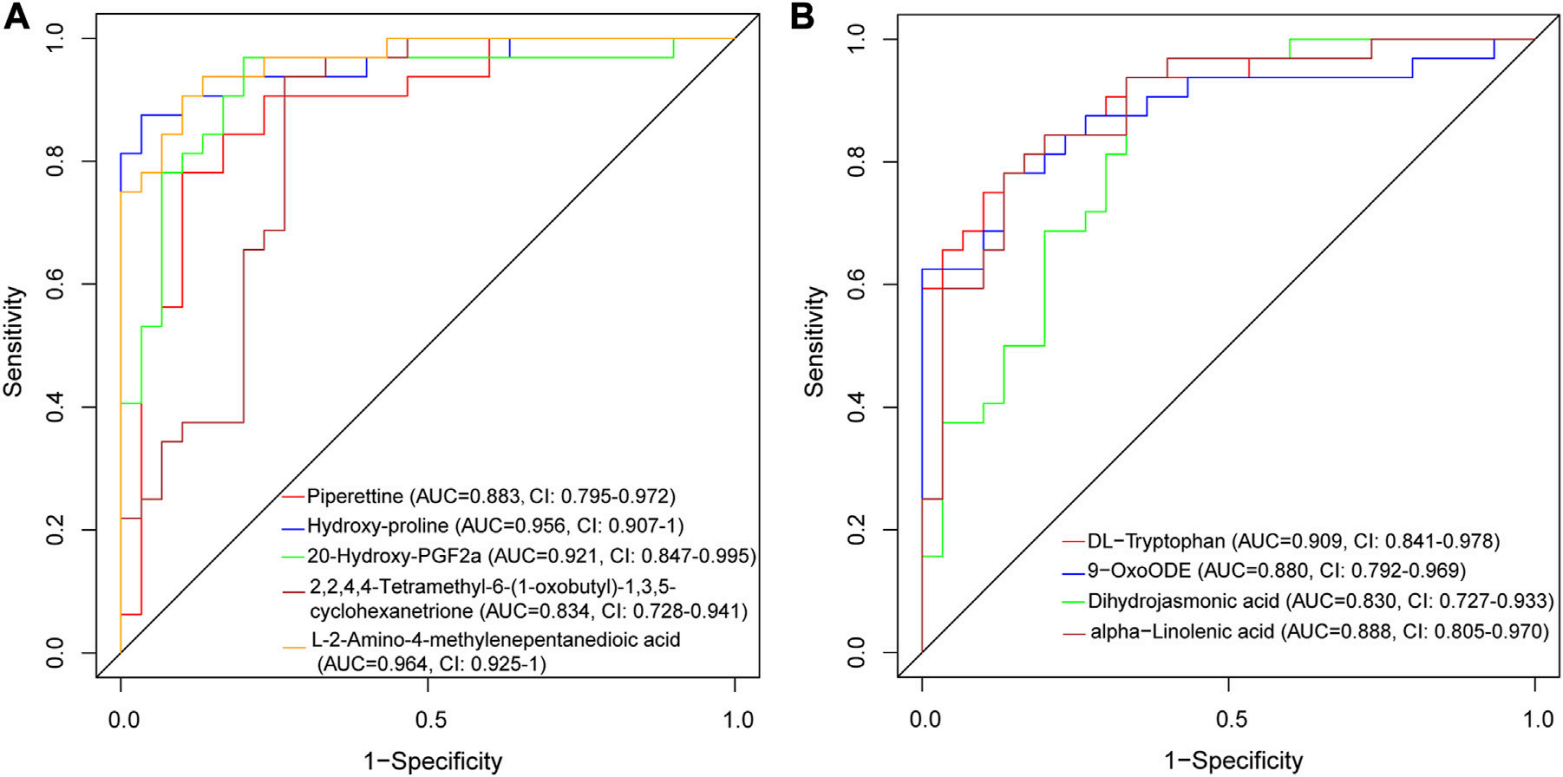
ROC curve of differentially expressed metabolites for AVM vs. HC group in positive ion mode **(A)** and negative ion mode **(B)**.

We evaluated the discriminative ability of the metabolites by plotting ROC curves for each metabolite and calculating the AUC. In the positive ion mode (Figure 7A), Piperettine (AUC = 0.883, CI: 0.795-0.972), hydroxy-proline (AUC = 0.956, CI: 0.907-1), L-2-Amino-4-methylenepentanedioic acid (AUC = 0.964, CI: 0.925-1), and 20-Hydroxy-PGF2a (AUC = 0.921, CI: 0.847-0.995) demonstrated high discriminative ability based on their AUCs. Similarly, in the negative ion mode (Figure 7B), DL-Tryptophan (AUC = 0.909, CI: 0.841-0.978), 9-OxoODE (AUC = 0.880, CI: 0.792-0.969), and alpha−Linolenic acid (AUC = 0.888, CI: 0.805-0.970) exhibited high discriminative ability.

## Discussion

AVMs are high-risk, complex and rare congenital vascular malformations that pose significant risks and challenges for treatment. However, current diagnostic methods lack specificity and effective experimental animal models for further research are lacking (Jones et al., 2021). To address this gap, untargeted metabolomics technology was used in this study to detect and analyze changes in plasma metabolites and metabolic pathway patterns in patients with extracranial AVM, with the goal of identifying AVM biomarkers and exploring their mechanism. As a result, 110 differential metabolites were screened in positive ion mode, of which 17 were upregulated and 93 were downregulated. In negative ion mode, 74 metabolites were identified, of which 19 were upregulated and 55 were downregulated. Lipids and lipid-like molecules were the most prevalent metabolites in both positive and negative ion modes. Major alterations to metabolic pathways were identified, including lipid metabolism, amino acid metabolism, carbohydrate metabolism, and protein translation in AVM patients compared to healthy controls. Through machine learning algorithms, we identified some characteristic metabolites for AVMs, including hydroxy-proline, L-2-Amino-4-methylenepentanedioic acid, piperettine, 20-hydroxy-PGF2a, 2,2,4,4-tetramethyl-6-(1-oxobutyl)-1,3,5-cyclohexanetrione in the positive ion mode, and DL-tryptophan, 9-oxoODE, alpha-Linolenic acid, dihydrojasmonic acid in the negative ion mode. Furthermore, the AUCs of the ROC curve indicated that hydroxy-proline, L-2-Amino-4-methylenepentanedioic acid, 20-Hydroxy-PGF2a, DL-Tryptophan, 9-OxoODE, and alpha−Linolenic acid had high discriminative ability as biomarkers for AVM disease. The results of this study provide new insights into the underlying pathogenesis of AVMs and offer potential new therapeutic targets for the disease. Overall plasma metabolomics may be a useful tool in developing objective biomarkers for identifying AVM and further research in this area is required.

Our study utilizing untargeted metabolomics demonstrates that lipid metabolism plays a crucial role in extracranial AVMs. We have identified 9-oxo-octadecadienoic acid (9-oxoODE) as a potential metabolite with promising diagnostic and predictive capabilities. This metabolite is produced from linoleic acid hydroperoxides via the LOX pathway and has previously been found to be elevated in the serum of patients with Alzheimer’s disease (Lu et al., 2017) and primary Sjogren’s syndrome (Han et al., 2022), as well as being a potential predictor of diabetic macular edema in older type 2 diabetes patients (Rhee et al., 2021). Furthermore, our study identified other significant metabolites that are components of the lipid metabolism pathway, such as alpha-Linolenic acid, dihydrojasmonic acid, and 20-hydroxy-PGF2a. Alpha-Linolenic acid is a type of omega-3 polyunsaturated fatty acid that has been associated with numerous health benefits, including cardiovascular disease prevention, diabetes management, cancer prevention, and improvement of cognitive function (Shahidi and Ambigaipalan, 2018). Dihydrojasmonic acid is a decomposition products of linolenic acid and acts as a plant growth regulator with similar physiological to prostaglandins in animals (Wasternack, 2007). PGF2a mainly acts on blood vessels and smooth muscle, participating in various physiological processes such as platelet aggregation, inflammation, pain, fever, nerve impulse transmission, and cell growth (Blackwell et al., 2010).

AVMs also have a significant impact on amino acid metabolism pathways compared to the healthy population. The differential pathways include aminoacyl-tRNA biosynthesis, D-glutamine and D-glutamate metabolism, lysine degradation, and lysine biosynthesis. The involved metabolites are DL-tryptophan and hydroxy-proline, which can affect protein metabolism. The enrichment of aminoacyl-tRNA biosynthesis pathways indicates increased protein translation in AVMs. Glutamine is a non-essential amino acid involved in various metabolic processes, such as energy production through the tricarboxylic acid cycle, synthesis of lipids and purines, and the production of the antioxidant compound glutathione (Curi et al., 2005). The metabolism of glutamine/glutamate is linked to immunomodulatory processes (Cruzat et al., 2018) and oxidative stress (Di Renzo et al., 2022). The supplementation of glutamine analogues can reduce endothelial injury (Herzog et al., 2020). This metabolic pathway also plays a role in the global disease COVID-19. A meta-analysis was performed on published metabolic data from COVID-19 patients, and of the 596 identified metabolites, pathway enrichment indicated that glutamine and glutamate metabolism was the most significant metabolic pathway (Li et al., 2023). DL-Tryptophan scavenges H2O2 and •OH more effectively than melatonin (Wang et al., 2013). However, a recent study (Cohen et al., 1979) suggested that DL-tryptophan might act as a tumor-promoting agent during bladder carcinogenesis. Proline is an important component of collagen and accounts for approximately 10% of all amino acids in collagen. The synthesis and degradation of lysine, one of the essential amino acids in the human body, have undergone changes over time. In a recent study (Marchesi et al., 2007), lysine levels were found to increase significantly in the feces of patients with inflammatory bowel disease. Lysine is metabolized to produce acetyl-CoA, which participates in the tricarboxylic acid (TCA) cycle. Glutamate can also be involved in the TCA cycle. This cycle is a crucial metabolic pathway in aerobic organisms and serves as the final metabolic pathway for carbohydrates, lipids, and amino acids. As a result, it organically connects the metabolism of these three major biomolecules together, functioning as the metabolic hub of energy. These observations are consistent with what is clinically observed, specifically that there is an increase in blood flow and skin temperature in AVM lesions. This suggests that the metabolism is more active than in normal tissue, and the metabolites may be present in peripheral blood as well.

The positive ion mode showed an enrichment of the caffeine metabolism pathway. Caffeine, a methylxanthine (1,3,7-trimethylxanthine), shares a similar molecular structure to adenosine and can bind to adenosine receptors, blocking the effects of adenosine and reducing fatigue and improving responsiveness (Fredholm et al., 1999). Moreover, caffeine may has been observed to protect against liver fibrosis by antagonizing adenosine receptor activation, which can promote tissue remodeling, including collagen production and fibrin generation. It may also protect against liver steatosis and fibrosis by improving fat homeostasis and reducing oxidative stress (Klemmer et al., 2011). In animal models, caffeine metabolites can reduce collagen deposition in hepatocytes and inhibit liver cancer occurrence (Hosaka et al., 2001). Furthermore, caffeine may improve energy balance by reducing appetite, increasing basal metabolic rate and food-induced thermogenesis (Harpaz et al., 2017). Caffeine may also affect pain transmission, neuroprotection, mood enhancement, and immune regulation, which may play a role in the occurrence of burning mouth syndrome. These mechanisms of caffeine could be relevant in AVMs by providing pain relief, reducing collagen and fibrin production, and reducing of oxidative stress to prevent fiber formation. The enrichment of the caffeine metabolism pathway in AVM suggests its potential role in AVM treatment. However, the roles of other distinct metabolites, including L-2-Amino-4-methylenepentanedioic acid, piperettine, and 2,2,4,4-tetramethyl-6-(1-oxobutyl)-1,3,5-cyclohexanetrione remain unknown.

Our study is subject to several limitations that need to be addressed. First, the sample size in this study is relatively small due to the rarity of the disease, making it challenging to gather sufficient samples. In addition, all of the patients in this study are from the same ethnic group and were recruited from a single study center, necessitating the need for cross-sectional and longitudinal studies involving more patients. Furthermore, the identification and analysis of all categories of metabolites simultaneously through non-targeted metabolomics is impractical due to various factors that influence the identification and analysis of metabolites, as well as the presence of numerous unknown metabolites in the metabolite database. Therefore, the differential metabolites identified in this study must be validated further in subsequent studies with a larger number of patients.

## Conclusion

In conclusion, this study demonstrated that patients with extracranial arteriovenous malformations exhibit significantly distinct metabolic profiles when compared to healthy individuals. By utilizing plasma metabolomics, it was possible to distinguish between extracranial AVM patients and the from healthy control population. These findings indicate that metabolomic analysis could aid in understanding the pathophysiology of AVMs and could serve as a potential tool for clinical diagnosis and treatment of the condition.

## Data Availability

The datasets presented in this study can be found in online repositories. The names of the repository/repositories and accession number(s) can be found below: MTBLS7753 (Metabolights).

## Supplementary material

SUPPLEMENTARY FIGURE S1 Presentation of QC samples in a 2-D PCA score plot. **(A)** Positive ion mode. **(B)** Negative ion mode.

SUPPLEMENTARY FIGURE S2 Presentation of QC Samples in a PCA One-Dimensional Profile. **(A)** Positive ion mode. **(B)** Negative ion mode.

SUPPLEMENTARY FIGURE S3 Pie chart of metabolite classification and proportion. **(A)** Pie chart of metabolite classification and proportion in positive ion mode. **(B)** Pie chart of metabolite classification and proportion in Negative ion mode. (Different color blocks in the figure represent different taxonomic categories, and the percentage represents the percentage of metabolites belonging to this type in the number of all identified metabolites).

SUPPLEMENTARY FIGURE S4 Score scatter plot of the PCA model for AVM vs. HC group in positive **(A)** and negative **(B)** ion mode.

SUPPLEMENTARY FIGURE S5 Heatmap of hierarchical clustering analysis for AVM vs HC. **(A)** Heatmap of hierarchical clustering analysis for all groups in positive ion mode. **(B)** Heatmap of hierarchical clustering analysis for all groups in negative ion mode. In the figure, the abscissa represents different sample groups, the ordinate represents all metabolites, the color blocks at different positions represent the relative expression number of metabolites at corresponding positions, red represents the expression of high content of the substance, and blue represents the expression of low content of the substance.

SUPPLEMENTARY FIGURE S6 Regulatory interaction network analysis for AVM vs HC. **(A)** Positive ion mode. **(B)** Negative ion mode. In the figure, the red dots represent metabolic pathways, the yellow dots represent substances related to regulatory enzyme information, and the green dots represent the background materials of metabolic pathway. The purple dots represent the molecular module information of class of substances, the blue dots represent chemical interactions of substances, and the green squares represent the different substances obtained by this comparison.
