# Supplementary figures and images for: Untargeted plasma metabolome identifies biomarkers in patients with extracranial arteriovenous malformations
Source: Front Physiol. 2023 Sep 1;14:1207390. doi: 10.3389/fphys.2023.1207390 (PMC10505742; doi:10.3389/fphys.2023.1207390)

A

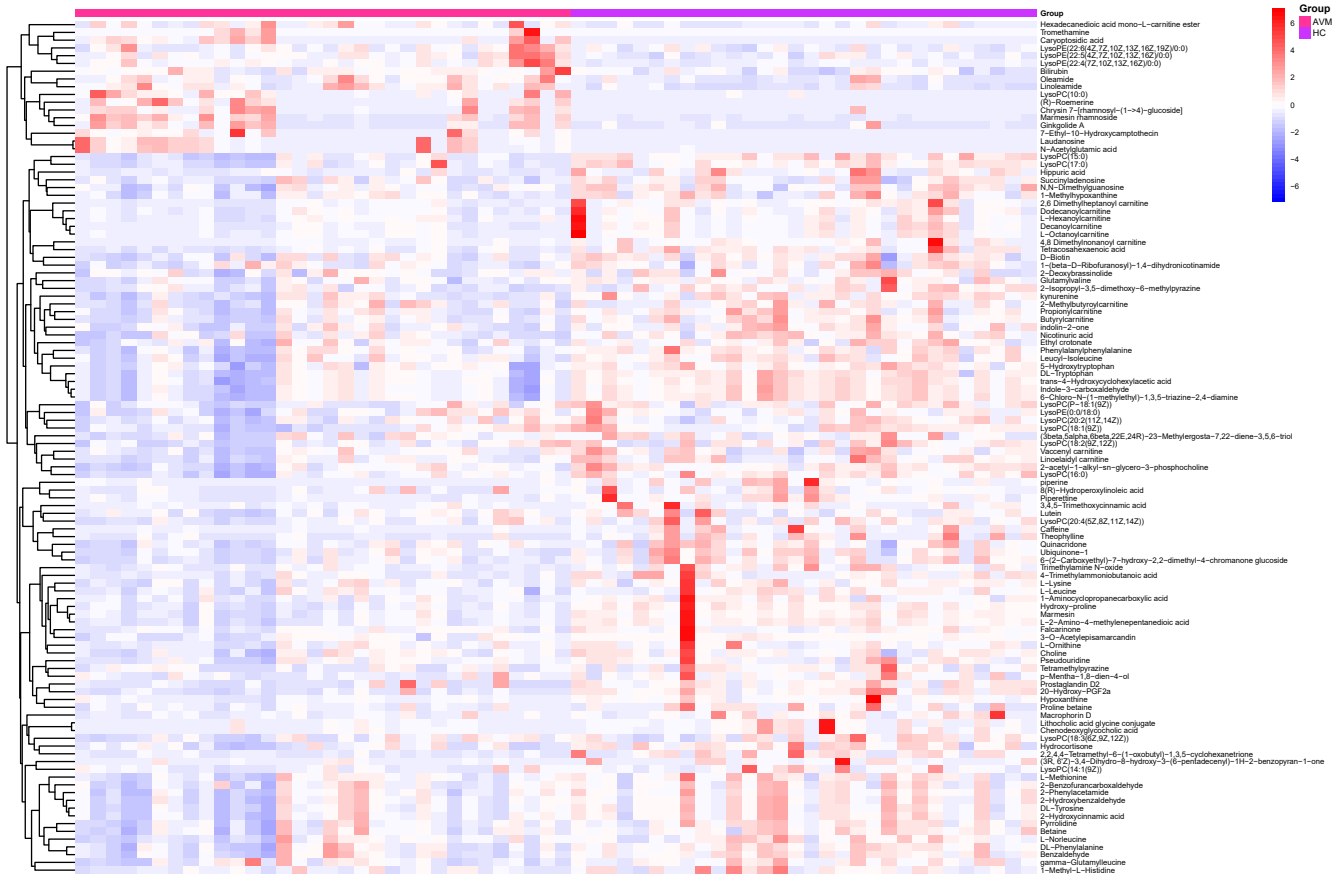

B

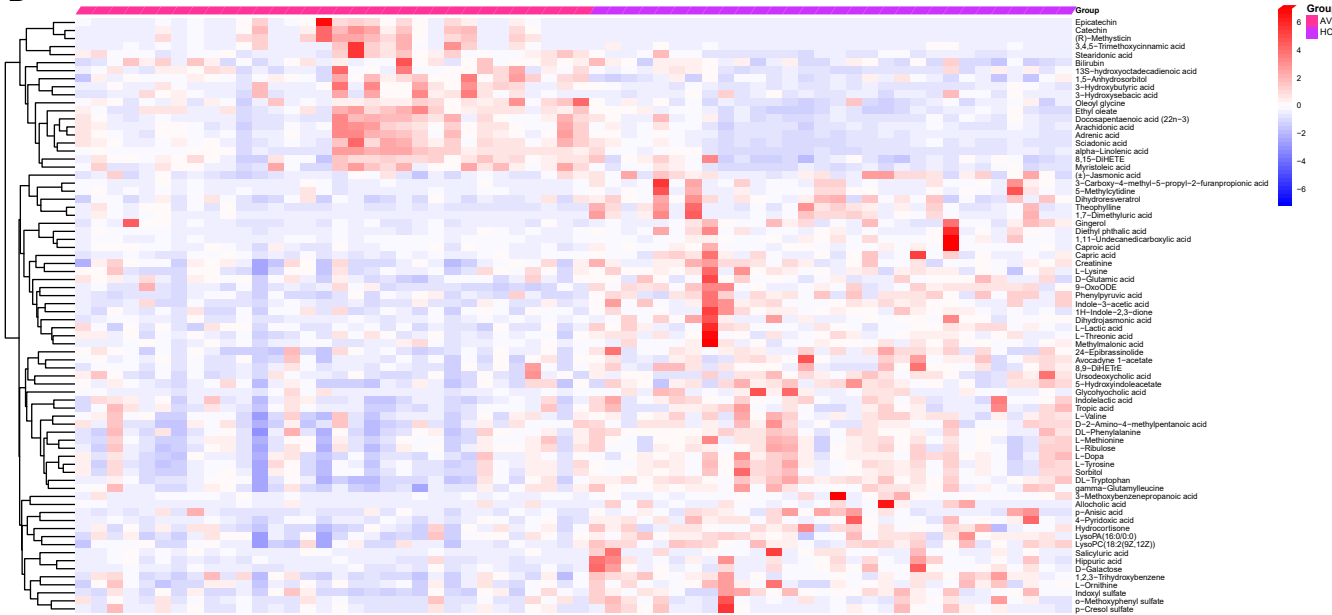

Supplement: Supplementary file 1 [file Image5.pdf]

A

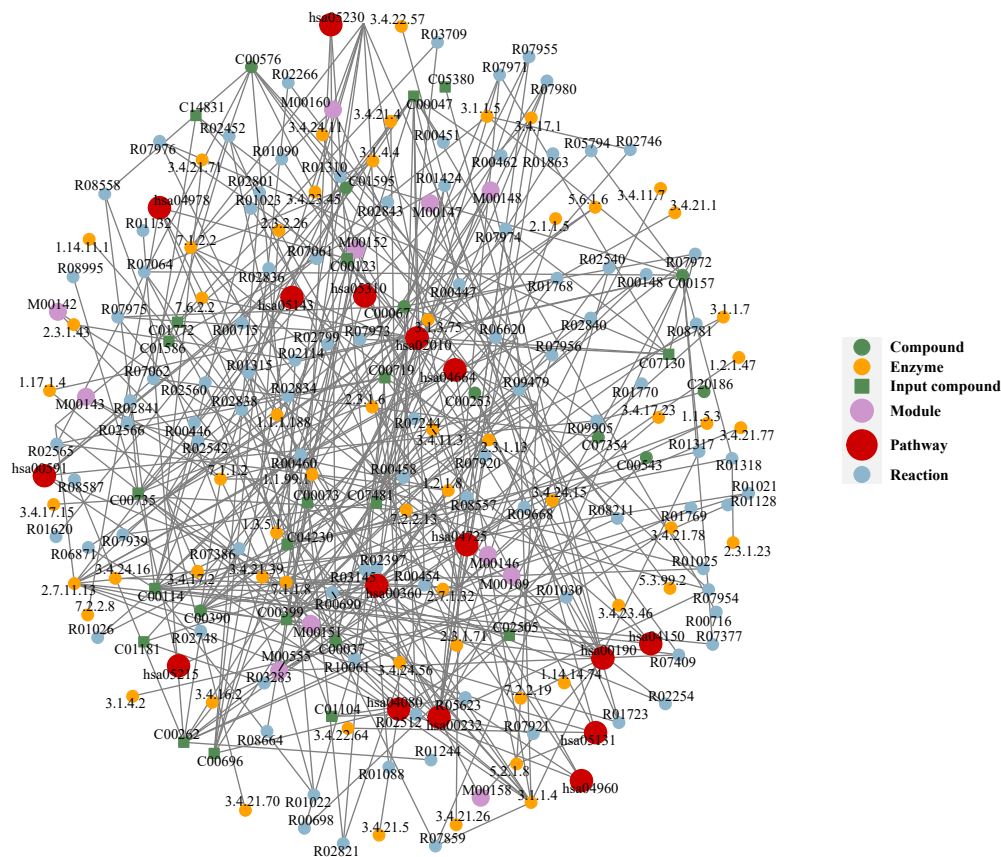

B

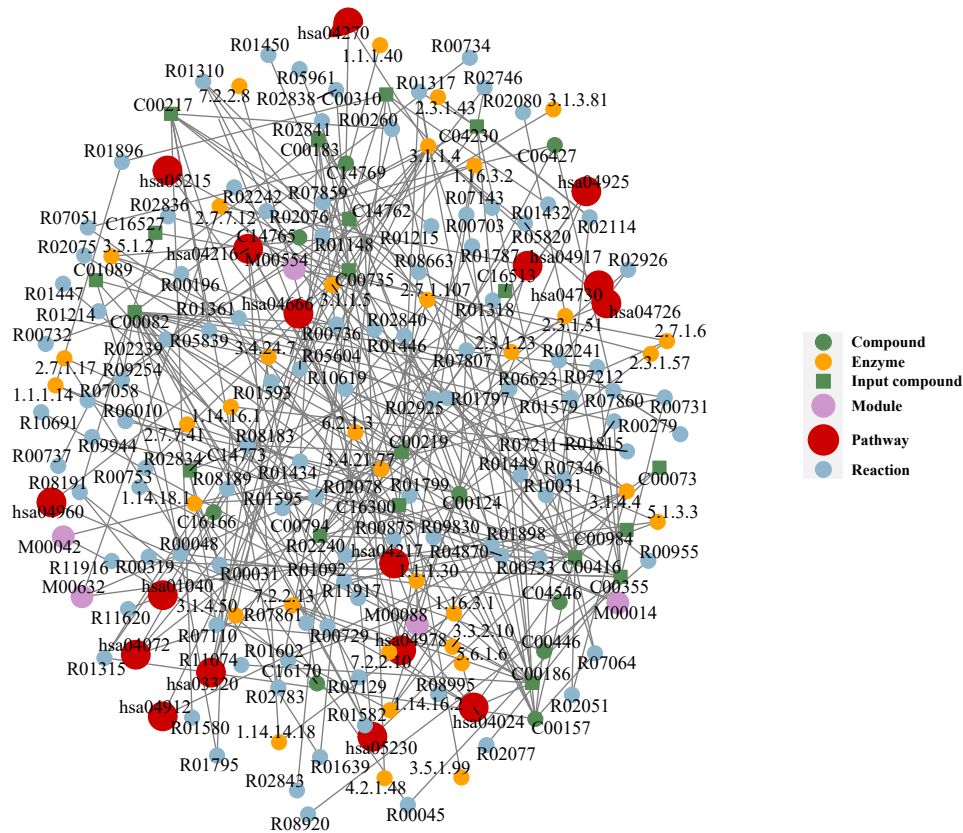

Supplement: Supplementary file 3 [file Image6.pdf]

A

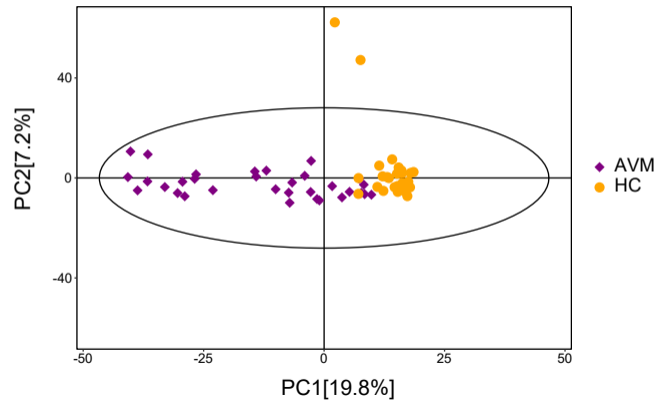

B

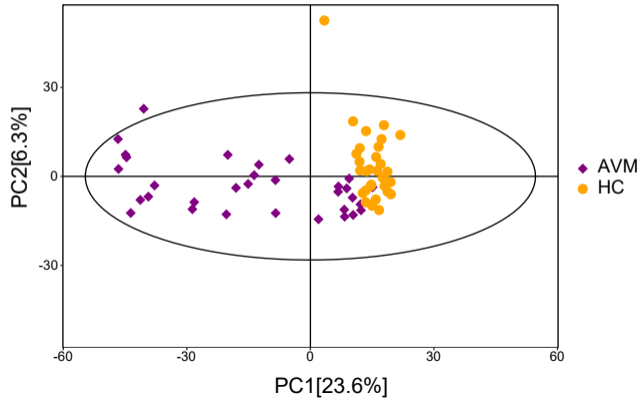

Supplement: Supplementary file 4 [file Image4.pdf]

A

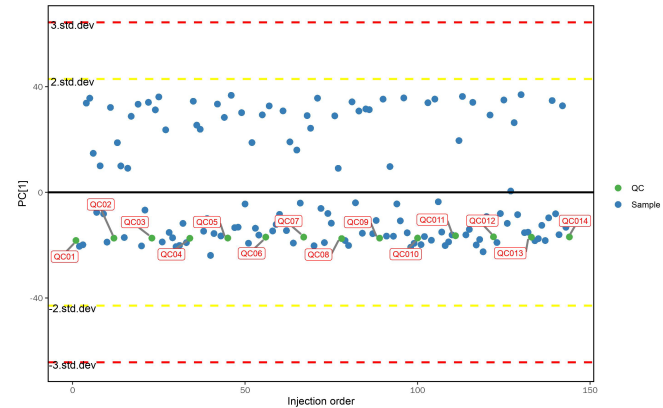

B

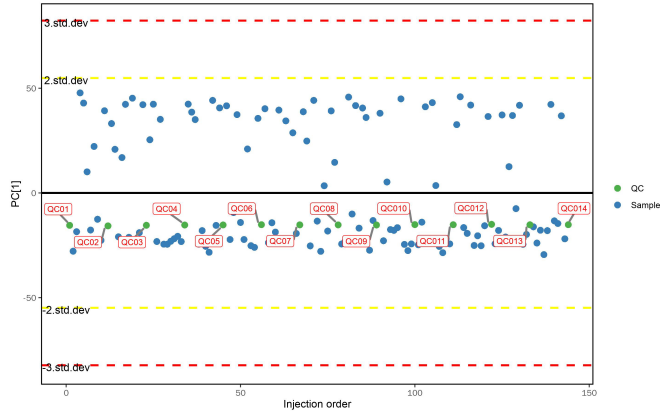

Supplement: Supplementary file 5 [file Image2.pdf]

A

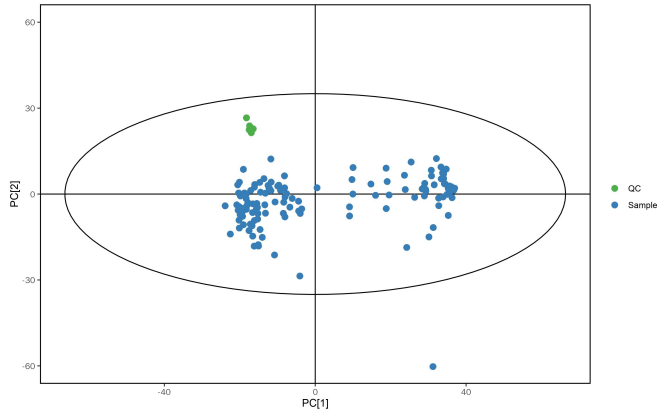

B

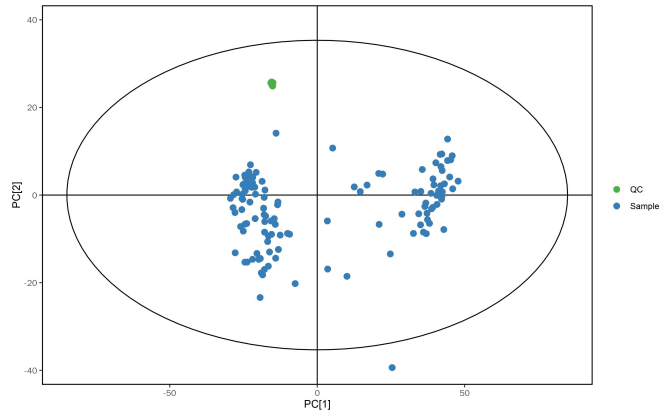

Supplement: Supplementary file 8 [file Image1.pdf]
