# Supplementary material for: Untargeted plasma metabolome identifies biomarkers in patients with extracranial arteriovenous malformations
Source: Front Physiol. 2023 Sep 1;14:1207390. doi: 10.3389/fphys.2023.1207390 (PMC10505742; doi:10.3389/fphys.2023.1207390)
Supplement: Supplementary file 2 [file Table1.docx]

Table S1. Search parameters used in the analysis

| Step | Parameter | Value/Range |
| --- | --- | --- |
| Data Preprocessing | Mass Error | ± 10 ppm |
|  | Retention Time (RT) Deviation | ± 0.5 min |
|  | XCMS Method | centWave |
|  | Peak Width | 5-20 seconds |
|  | Signal-to-Noise (SN) | 3 |
|  | Pre-filtering Step | Intensity ≥ 1000(minimum 3 values) |
| Feature Selection | Variable Importance Threshold | ≥1 |
|  | P-value Threshold | ≤ 0.05 |
| Metabolite Annotation | Database Matching | HMDB, KEGG, etc. (in priority order) |
|  | Mass Accuracy | ≤ 5 ppm |
|  | Fragment Ion Match Score | ≥ 80% |
|  | Minimum m/z difference for overlapping retention time peaks | -0.001 |
