# Supplementary material for: Untargeted plasma metabolome identifies biomarkers in patients with extracranial arteriovenous malformations
Source: Front Physiol. 2023 Sep 1;14:1207390. doi: 10.3389/fphys.2023.1207390 (PMC10505742; doi:10.3389/fphys.2023.1207390)
Supplement: Supplementary file 6 [file Image3.pdf]

A

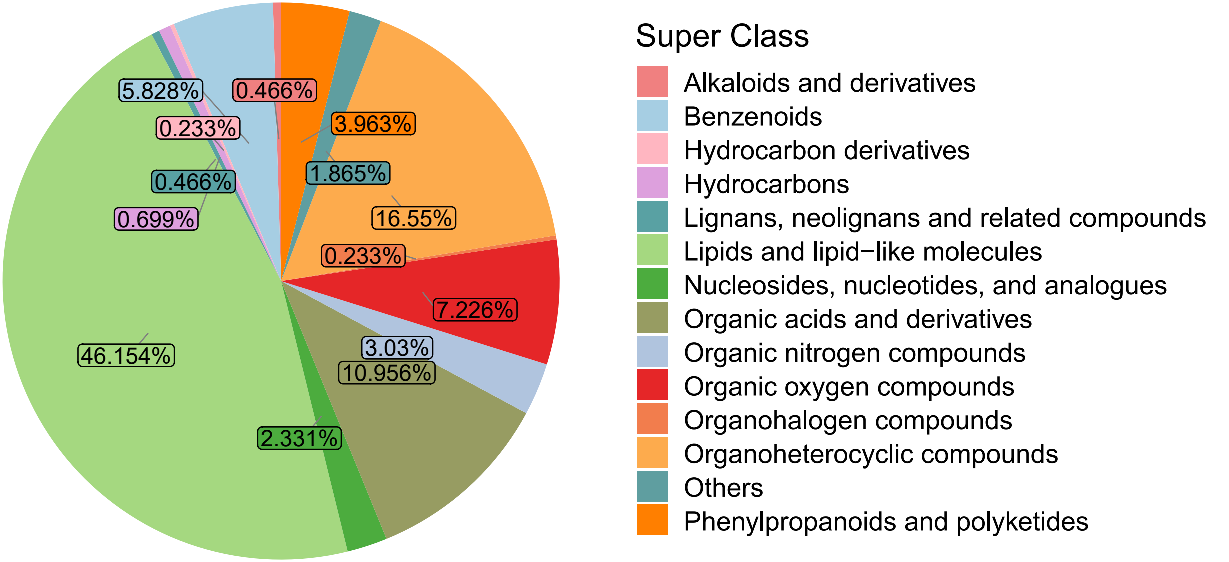
5.828%
0.466%
0.233%
3.963%
1.865%
0.466%
0.699%
16.55%
0.233%
7.226%
46.154%
3.03%
10.956%
2.331%
Super Class
Alkaloids and derivatives
Benzenoids
Hydrocarbon derivatives
Hydrocarbons
Lignans, neolignans and related compounds
Lipids and lipid−like molecules
Nucleosides, nucleotides, and analogues
Organic acids and derivatives
Organic nitrogen compounds
Organic oxygen compounds
Organohalogen compounds
Organoheterocyclic compounds
Others
Phenylpropanoids and polyketides

B

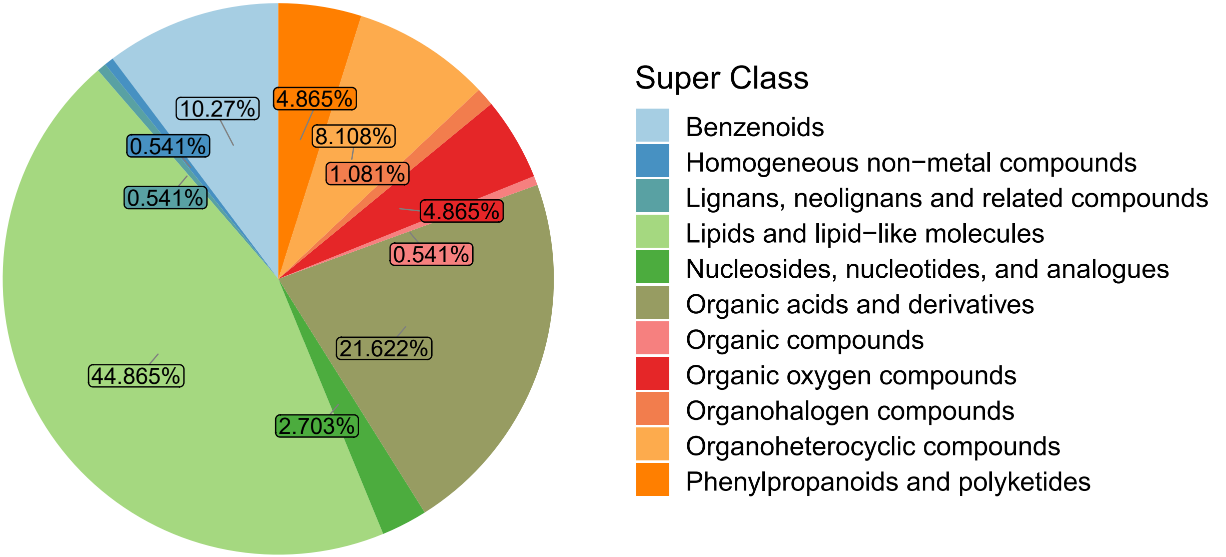
10.27%
4.865%
8.108%
0.541%
1.081%
0.541%
4.865%
0.541%
21.622%
44.865%
2.703%
Super Class
Benzenoids
Homogeneous non−metal compounds
Lignans, neolignans and related compounds
Lipids and lipid−like molecules
Nucleosides, nucleotides, and analogues
Organic acids and derivatives
Organic compounds
Organic oxygen compounds
Organohalogen compounds
Organoheterocyclic compounds
Phenylpropanoids and polyketides
