# Supplementary material for: Untargeted plasma metabolome identifies biomarkers in patients with extracranial arteriovenous malformations
Source: Front Physiol. 2023 Sep 1;14:1207390. doi: 10.3389/fphys.2023.1207390 (PMC10505742; doi:10.3389/fphys.2023.1207390)
Supplement: Supplementary file 7 [file Table2.docx]

Table S2. Clinical characteristics of subjects.

| Varieties | AVM (n=32) | HC (n=30) | P-value |
| --- | --- | --- | --- |
| Age (years), mean ± SD | 35.78±12.62 | 38.63±11.77 | 0.362 |
| Gender (male/female), n | 14/18 | 18/12 | 0.201 |
| BMI (kg/m^2^), mean ± SD | 23.53±3.94 | 23.91±4.14 | 0.711 |
| Accompanying diseases |  |  |  |
| Hypertension (yes/no), n | 3/29 | 2/28 | 0.696 |
| Diabetes (yes/no), n | 0/32 | 0/30 | 1 |
| Hyperlipidemia (yes/no), n | 1/31 | 1/29 | 0.963 |
| Autoimmune diseases (yes/no), n | 0/32 | 0/30 | 1 |
| Liver/heart/kidney failure (yes/no), n | 0/32 | 0/30 | 1 |
| Malignant tumor (yes/no), n | 0/32 | 0/30 | 1 |
